# Side effects following vaccination in multiple sclerosis: a prospective, multi-centre cohort study

**DOI:** 10.1038/s41598-023-41271-6

**Published:** 2023-09-02

**Authors:** Alexander Winkelmann, Christoph Metze, Uwe K. Zettl, Micha Loebermann

**Affiliations:** 1 Department of Neurology, University of Rostock, Gehlsheimer Strasse 20, 18147 Rostock, Germany; 2 Neuroimmunological Section, Department of Neurology, University of Rostock, Gehlsheimer Strasse 20, 18147 Rostock, Germany; 3 Kliniken im Theodor-Wenzel-WerkKlinik für Psychiatrie, Potsdamer Chaussee 69, 14129 Berlin, Germany; 4 Department of Tropical Medicine and Infectious Diseases, University of Rostock, Ernst Heydemann Strasse 6, 18059 Rostock, Germany

**Keywords:** Outcomes research, Multiple sclerosis, Inactivated vaccines

## Abstract

Vaccines play a crucial role in preventing infections in patients with multiple sclerosis (MS), although concerns have been raised about potential worsening of the underlying disease. To investigate this, we conducted a prospective, multicentre, non-randomized observational study assessing changes in disease activity, safety, and clinical tolerability of vaccination in 222 MS patients on disease-modifying drugs. The majority of patients were female (76.6%) and 89.6% had relapsing–remitting MS. The vaccines administered were primarily seasonal influenza (56.3%) or tetanus-based vaccines (33.8%). Disease activity, as measured by annualized relapse rate, decreased significantly from 0.64 the year prior to vaccination to 0.38 in the following year. Moreover, the extended disability status scale remained stable within six months after vaccination in comparison to pre-vaccination values. Side effects were reported in 19.2% of vaccinated subjects, most commonly local side effects (65.2%) or flu-like symptoms (34.8%). Our findings suggest that standard non-live vaccines are safe and well-tolerated in MS patients and do not negatively impact disease activity.

## Introduction

Preventive vaccination plays an important role in the management of health issues, especially when caring for people with multiple sclerosis (MS)^1,2^. Infectious episodes and fever may lead to the deterioration of MS disease contributing to increased mortality rates compared to the normal population^3^. Prevention of infections is an important measure of medical care for patients with MS^4,5^. Patients and treating physicians have previously been concerned about reports of possible detrimental effects of vaccinations on the course of MS^6,7^. This may still lead to vaccine hesitancy in MS patients despite studies revealing that non-live vaccines are safe and treatment guidelines recommend consequent vaccination in these patients^8,9^.

The aim of this study was to evaluate the safety, tolerability, and MS disease activity following generally recommended vaccines in MS in a real-world situation.

## Patients, material and methods

This prospective, multicentre, non-randomized observational study at specialized outpatient MS care centres included MS patients aged 18 to 70 years who had been on disease-modifying treatment (DMT) for at least six months and who had an indication for recommended preventive vaccination^10^. We excluded all patients with MS relapse or other disease activity during the previous six months before vaccination. Routine MRI evaluation was not planned in this study protocol.

Participation in this study was offered to all patients who received licensed vaccinations on a routine basis. Details of MS disease, medical history, baseline characteristics and clinical examination were collected. Subjects received the respective vaccination in the deltoid muscle in an open-label manner. Subjects had follow-up visits after 1, 3, 6 and 12 months between 2010 and 2013. Local and systemic adverse events were registered at the first follow-up visit 4 weeks after vaccination. Data of pre-vaccination disease activity was collected from clinical records in a retrospective manner. MS relapse activity as well as expanded disability status scale (EDSS) after vaccination were monitored throughout follow-up visits prospectively. Relapses were confirmed by the treating neurologists and medical evaluation according to national guidelines following routine patient management and medical practice.

The evaluation of the immune response to influenza and tick-born encephalitis (TBE) vaccines of patients presented in this study have previously been published^11,12^.

All statistical analyses were performed using Prism 9 (9.1.2, GraphPad Software). Values were expressed as mean ± standard deviation. Mann–Whitney U-test was used to compare annualized relapse rates. All reported p-values are two-sided; values of 0.05 or less were considered to indicate statistical significance.

The study was approved by the local ethics committee (Rostock HV 2010-0002) and registered at ClinicalTrials.gov (NCT02275741). The study was conducted in accordance with the International Conference on Harmonization Guidelines for Good Clinical Practice, the Declaration of Helsinki and all applicable national laws. Informed written consent was obtained from all participants before entry into the study.

## Results

In total 222 patients with MS were included in this analysis. The baseline characteristics of this cohort are summarized in Table 1. The majority of included subjects were female (76.6%), and most of the patients were affected by RR-MS (89.6%) and had a mean duration of disease of 8.5 ± 6.9 years. The mean EDSS score at vaccination was 2.3 ± 1.9.

**Table 1:** Characteristics of the enrolled subjects.

	Total (N = 222)
Age (years) ± SD	41.32 ± 10.63
Sex	
Male	52 (23.4%)
Female	170 (76.6%)
Mean duration of disease ± SD (years)	8.5 ± 6.9 (min 0.2; max 33.4)
Mean EDSS at vaccination ± SD	2.3 ± 1.9 (min 0; max 8.5); n = 220
MS disease course	
RR-MS	199 (89.6%)
SP-MS	13 (5.9%)
PP-MS	4 (1.8%)
Unknown	6 (2.7%)
Current DMD at vaccination	
Interferon beta	106 (47.8%)
Interferon beta 1b	31 (14.0%)
Interferon beta 1a s.c	49 (22.1%)
Interferon beta 1a i.m	26 (11.7%)
Glatiramer acetate	52 (23.4%)
Teriflunomide	1 (0.5%)
Fingolimod	13 (5.9%)
Natalizumab	26 (11.7%)
IVIg	2 (0.9%)
Cyclic GCS pulse	7 (3.2%)
Azathioprine	1 (0.5%)
Mitoxantrone	3 (1.2%)
Triamcinolone, intrathecal	1 (0.5%)
No treatment	9 (4.1%)
Unknown (study medication)	1 (0.5%)
Vaccination	
Influenza (seasonal trivalent inactivated)	125 (56.3%)
Tetanus-based vaccines (adsorbate)	75 (33.8%)
Td	12 (5.4%)
Tetanus	10 (4.5%)
Td aP	14 (6.3%)
Td aP IPV	25 (11.3%)
Td IPV	14 (6.3%)
Poliomyelitis (IPV)	1 (0.5%)
Tic borne encephalitis (inactivated)	20 (9.0%)
Hepatitis unspecified	4 (1.8%)
Hepatitis B (recombinant HBs-antigen)	6 (2.7%)
Hepatitis A (inactivated)	4 (1.8%)
Hepatitis A/hepatitis B	2 (0.9%)
Typhoid fever (Vi-antigen)	2 (0.9%)
Pneumococcal (polysaccharide)	3 (1.2%)
Rubella (live attenuated)	1 (0.5%)
Cholera (inactivated)	1 (0.5%)

Simultaneous vaccinations were possible.

*aP* acellular pertussis, *DMD* disease modifying drug, *GCS* glucocorticosteroids, *IPV* inactivated poliomyelitis virus vaccine, *IVIg* intravenous immunoglobulin, *PP-MS* primary progressive multiple sclerosis, *RR-MS* relapsing–remitting multiple sclerosis, *SD* standard deviation, *SP-MS* secondary progressive multiple sclerosis, *Td* tetanus/diphtheria.

The majority of subjects received interferon-beta therapy (47.8%) at vaccination; other applied DMTs were glatiramer acetate (23.4%), natalizumab (11.7%), fingolimod (5.9%). Of the subjects 56.3% received seasonal influenza vaccination, 33,8% received tetanus-based vaccination, 9.0% had a TBE vaccination. The other subjects were vaccinated against various other transmittable diseases, among them hepatitis A or B and typhoid fever.

**Figure 1:**
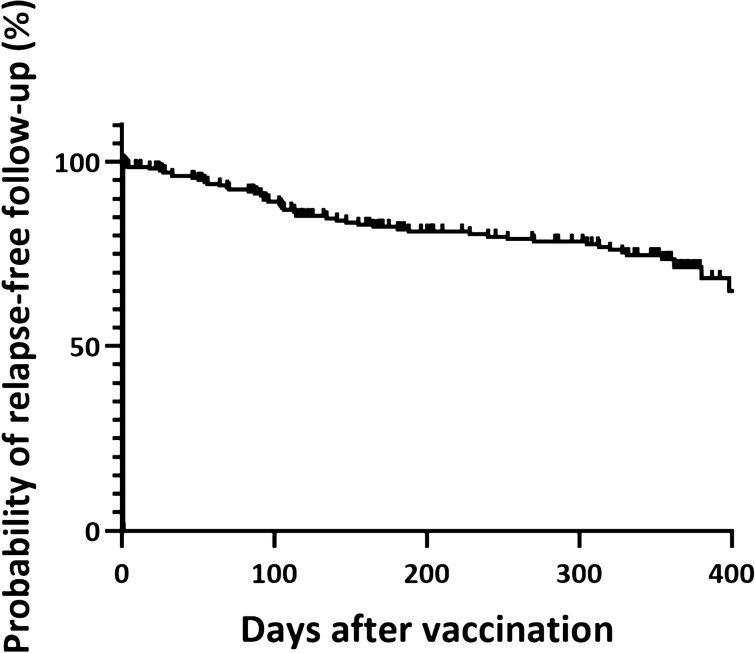
Relapse-free follow-up after vaccination. Graph (Kaplan–Meier plot) shows the probability of relapse-free follow-up after vaccination. Each reduction represents the occurrence of a relapse episode over time in the studied population.

After vaccination 6 subjects had a relapse in the first month (3.3%), among those, 3 (1.6%) subjects within the first week, 1 (0.5%) during the third week and 2 (1.1%) during the fourth week. Within the second and third month after vaccination 6 (3.3%) and 5 (2.7%), respectively had relapses (Fig. 1).

Over all, 14 relapses during day 7 and 90 following vaccination (7.6%) were reported. Between day 7 and 90 after vaccination relapses occurred in 9/125 (7.2%) patients after influenza, 4/75 (5.3%) after tetanus-based, and 1/20 (5.0%) after tic borne encephalitis vaccination, resulting in annualized relapse rates of 0.29, 0.21, and 0.2 respectively.

In 8/106 (7.6%) patients treated with interferon-beta and in 3/52 (5.8%) patients treated with glatiramer acetate relapses occurred between day 7 and 90 after vaccination, resulting in annualized relapse rates of 0.30 and 0.23, respectively.

**Figure 2:**
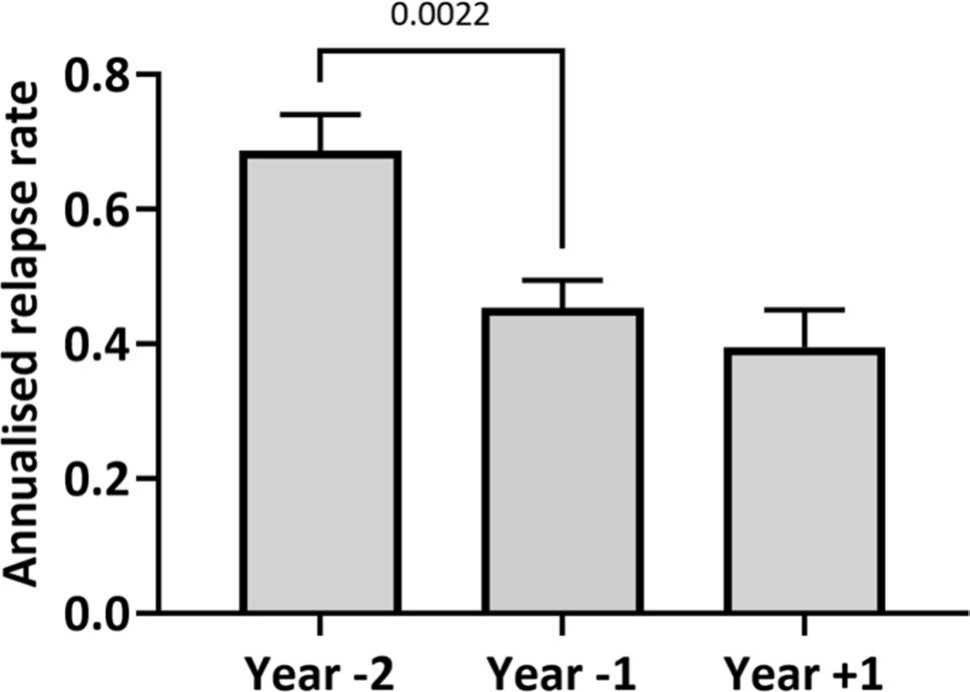
Annualised relapse rate. Mean annualised relapse rates in the study population during 2 years before vaccination and 1 year after vaccination, error bars show standard error of means. Means between year −1 and year +1 are not significantly different, p (year −1 vs. year +1) is > 0.05 (Mann–Whitney U test).

The annualized relapse rate decreased from 0.69 to 0.45 during the 2 years before vaccination (p < 0.05). In the year following vaccination the annualized relapse rate (0.4) was not significantly changed (p > 0.05) compared to the preceding year (Fig. 2).

**Figure 3:**
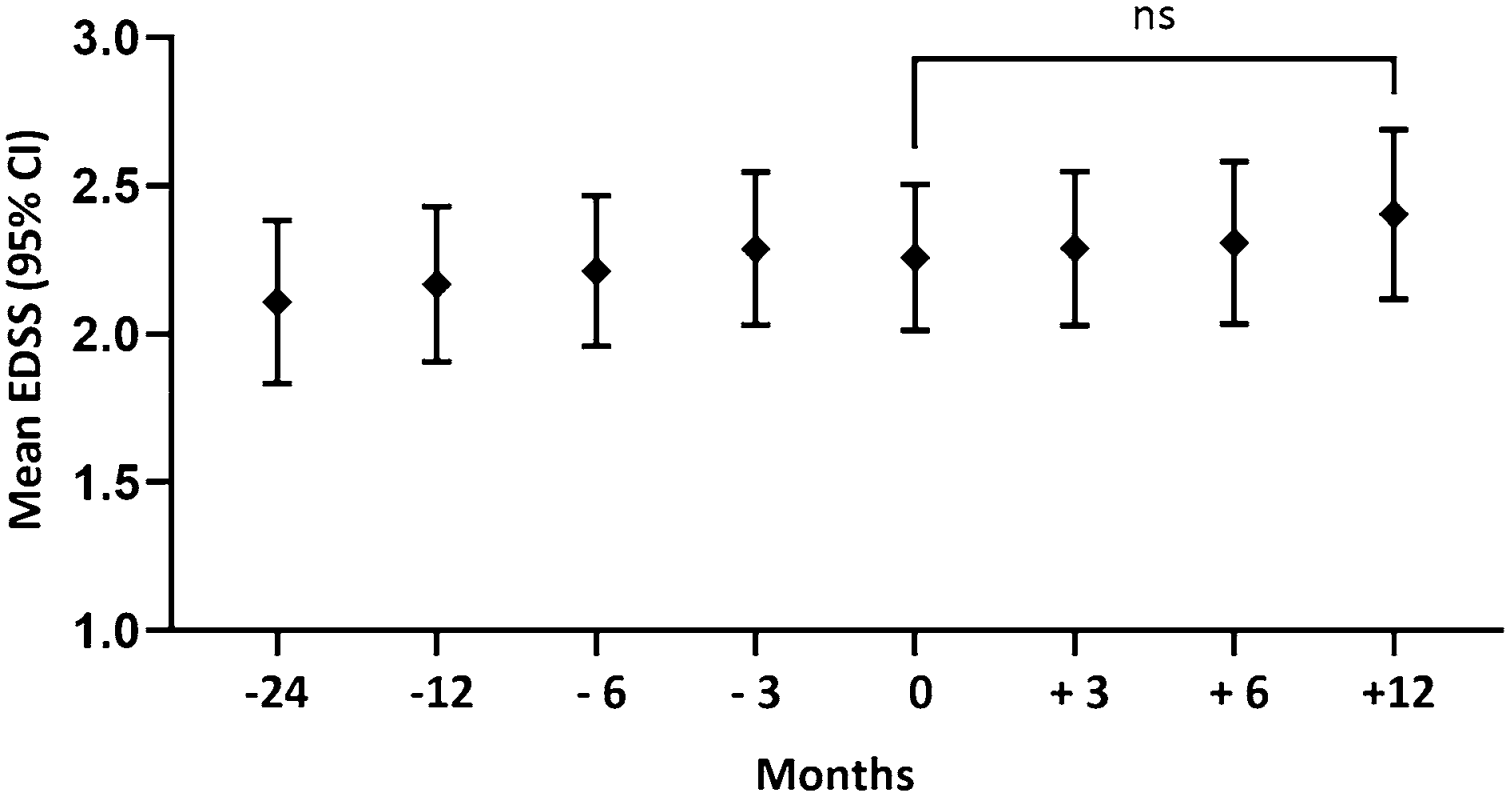
Mean Expanded Disability Status Scale (EDSS) during 24 months before vaccination and 12 months after vaccination. Whisker bars indicate 95% confidence interval. *ns* not significant.

EDSS score was not significantly different during 24 months before vaccination and within 6 months after vaccination (Fig. 3). Over all the EDSS was higher, though not significantly changed, 12 months after vaccination (2.4) compared to the EDSS at vaccination (2.3, p = 0.35).

Adverse events following vaccination were documented in 40 patients (20.2%), among them 24 reported local and 21 reported systemic adverse events (Table 1). Most of the reported adverse effects were mild and subsided within the first week. Except for local pain at the injection site that was rated as severe in one subject, all other local side effects were mild. Flu-like symptoms were the most commonly reported systemic side effects, of these 2 (13.3%) were severe, 7 (46.7%) were moderate and 6 (40.0%) mild (Table 2).

**Table 2:** Side effects following vaccination.

	N	%
Local adverse events	24	12.1
Pain at injection site	14	7.1
Swelling	8	4.0
Soreness	2	1.0
Induration	3	1.5
Erythema	6	3.0
Systemic adverse events	21	10.6
Flu-like symptoms	15	7.6
Fatigue	3	1.5
Generalized exanthema	1	0.5
Headache	2	1.0

Following influenza vaccination 12/125 (9.6%) had local and 13/125 (10.4%) had systemic adverse events, among them three patients had both, local and systemic side effects. In tetanus-based vaccine recipients 11/75 (14.7%) had local and 6/75 (8.0%) had systemic adverse events, among those, one patient had both, local and systemic adverse events. Following tick-borne vaccination 3/20 (15.0%) had local side effects, 2/20 (10.0%) had systemic reactions and one had both, local and systemic reactions.

In patients treated with interferon-beta 14/106 (13.2%) had local and 12/106 (11.3%) had systemic adverse events, among them two patients had both, local and systemic side effects.

In patients treated with glatiramer acetate 9/52 (17.3%) had local and 5/52 (9.6%) had systemic adverse events, among them three patients had both, local and systemic side effects.

## Discussion

The fear of possible adverse effects of vaccination in general and specific to people with MS, like increased relapse activity and progression (EDSS), might occasionally lead to vaccine hesitancy in MS patients and their physicians^7,13–16^.

The focus of this study was to analyse possible MS specific effects and other adverse events following vaccination. Since MS develops changes in long term, we have evaluated subjects with MS during one year before and after vaccination. This enables to determine changes in relapse-rate and EDSS and may provide a better insight into relapse timing and possible association to vaccination in a real-world situation.

The analyzed real-world study population represents a cohort of out-patients with MS suitable for vaccination in a typical gender and age distribution^17–19^. The studied population had stable disease with annualised relapse rates of 0.69 and 0.45 during the 2 years before vaccination. Notably, the annualised relapse-rates were high in this study population before vaccination. This might reflect a selection bias towards people with MS and higher disease activity treated at the involved specialised MS clinics^19–21^. The reduction of the relapse reflects the effects of the established and ongoing DMT^22–24^. In the year following vaccination the annualized relapse rate (0.4) was not significantly changed compared to the time before vaccination. This implies, that routine vaccination does not negatively impact the annualized relapse rates in MS patients which has been shown in in previous studies as well^25,26^. One has to keep in mind that only inactivated, non-live vaccines have been used in our study. Other studies have hinted a possible increase in relapse rates after live vaccines, such as yellow fever^13^, though this was not confirmed in a recent study^27^.

The EDSS, a marker of disease progression in MS that is rarely reported in the context of vaccination, showed a mild over-all increase during the study period of 3 years, corresponding to a MS cohort with mean disease duration of 8.5 years^28,29^. During 12 months after vaccination the EDSS did not significantly change in comparison to the EDSS at vaccination.

Local and systemic adverse events of vaccination in the general population are extensively studied before licensing of vaccines and are generally assessed using subject diaries and short term follow-up visits in vaccination studies. This study did not focus on short-lived expected adverse events and thus did not use diaries, but relied on reporting of adverse events at close and structured follow-up visits. Therefore one may expect a reporting bias, since minor side effects may be underreported at retrospective documentation^30,31^. Nevertheless, the reported adverse events were rare and generally mild. Should severe cases of adverse events have occurred one may expect, that these events would have been reported despite a possible recall bias.

On the one hand this prospective multicentre study shows that vaccine side effects in patients with MS do not present any particular quantitative or qualitative safety signals compared with the general population. On the other hand, the disease activity of MS is not negatively affected by non-live vaccines. This also seems true for novel mRNA-based SARS-CoV2 vaccines in patients with MS^32–36^, though long-term evaluations are pending^14^.

We feel this study may contribute to overcome possible vaccine hesitancy in MS patients and their treating physicians^37,38^ by documenting low rates of adverse events following-routine vaccinations comparable to the expected spectrum of side effects in the general population.

## Data Availability

The datasets used and/or analysed during the current study are available from the corresponding author on reasonable request.
